# Anesthesia depth monitoring during opioid free anesthesia – a prospective observational study

**DOI:** 10.1186/s12871-024-02859-1

**Published:** 2025-01-24

**Authors:** Krister Mogianos, Anna KM Persson

**Affiliations:** 1 Department of Anesthesiology and Intensive Care Medicine, Halland Hospital Halmstad, Lasarettsvägen, Halmstad, SE-30581 Sweden; 2 Department of Clinical Sciences Malmö, Lund University, Malmö, Sweden

**Keywords:** Processed electroencephalography, Opioid-free anesthesia, Opioid-based anesthesia, Density spectral array, Spectral edge frequency, Patient state index

## Abstract

**Background:**

Patients undergoing general anesthesia are more frequently monitored for depth of anesthesia using processed electroencephalography. Opioid-free anesthesia is nowadays an accepted modality for general anesthesia, however it is unclear how to interpret data from processed electroencephalography when using a mixture of non-opioid anesthetic drugs. Our objective was to describe density spectral array patterns and compare processed encephalographic data indices between opioid-free and routine opioid based anesthesia.

**Methods:**

This prospective observational cohort study was conducted on 30 adult patients undergoing laparoscopic surgery in a non-tertiary regional hospital. The patients underwent general anesthesia with three different methods and were monitored for anesthesia depth using processed encephalography and density spectral array. Primary outcome is a group-derived mean difference in patient state index and spectral edge frequency. As a secondary outcome a descriptive comparison of the spectral power, derived from the density spectral array, was done between groups.

**Results:**

The opioid-free anesthesia group had significantly higher patient state index and spectral edge frequency compared to routine anesthesia. Density spectral array patterns were also different, most notably lacking the high power in alpha frequency spectrum seen in the other routine anesthesia methods.

**Conclusions:**

Processed electroencephalography monitoring can be used in opioid-free anesthesia, however clinicians should expect higher values in monitoring indices. The density spectral array pattern using a common protocol for opioid-free anesthesia, with mainly sevoflurane combined with low doses of dexmedetomidine and esketamine, differs from well described opioid and GABA-ergic anesthesia methods. These findings should be further validated using other protocols for opioid-free anesthesia in order to safely monitor anesthesia depth.

**Trial registration:**

Clinicaltrials.gov registration number NCT06227143, registration date; 26th of January 2024.

## Background

Providing opioid free anesthesia (OFA) for major surgery has during the past decade proven to be both feasible and safe and has shown good results on postoperative pain outcome, without the side effects of opioids [1, 2]. The key concept in this anesthesia method is combining well-known anesthetic agents, which act synergistically in low doses to produce the desired effect, i.e. hypnosis, antinociception, amnesia, immobilization and neuromuscular blockade without compromising the hemodynamic physiology [3]. However, for anesthesia providers trained mainly in opioid-based anesthesia this is a challenging task. Some clinical signs, usually used for determining adequate level of anesthesia, are lacking, making assessment challenging.

Processed electroencephalography (pEEG) is one adjunct available to anesthesia providers when assessing brain function during anesthesia. Typically, pEEG contains unprocessed raw-EEG and an algorithm-based index-value between 0 and 100 for example the patient state index (PSI) (Massimo™) [4] and the spectral edge frequency under 95% (SEF95), a point in the frequency spectrum below which 95% of the total spectral power is contained [5]. Processed EEG is a way of monitoring brain function to determine anesthsia depth in patients under general anesthesia [6, 7]. Since its discovery this monitoring method has been refined into commercially available devices. By using pEEG in clinical practice, anesthesia providers have developed empirical knowledge of how to use this technology when assessing brain function to determine anesthesia depth. However, this is only true for routine anesthesia protocols using volatile agents or propofol together with opioids.

Since real-time assessment of unprocessed raw-EEG patterns requires profound knowledge and experience in order to identify disturbances in brain function during anesthesia, simplifying methods have been implemented in monitoring devices. Density spectral array (DSA) is a color-coded spectrogram, containing the power of exhibited frequencies over time [8, 9] enabling easy interpretation of where the patient´s current oscillating frequencies are focused for the cortical area monitored [6].

Density spectral array is useful as an adjunct in clinical practice. However, it requires knowledge on how specific anesthetic regimens look at different brain states during general anesthesia. Current evidence fails to present a guide on how to interpret pEEG in new types of anesthesia methods where anesthetic drugs are combined, like in OFA. Therefore, the aim of this prospective cohort-study was to evaluate pEEG, specifically PSI, SEF95 and DSA, during OFA and compare the values to those seen during routine anesthesia with either volatile anesthetic or total intravenous anesthesia (TIVA) in conjunction with opioids. We hypothesized that PSI and SEF95 for OFA will be higher, for the same anesthesia depth, compared to routine anesthesia methods and that DSA-pattern differed between the anesthetic regimens.

## Methods

### Patient recruitment and ethics

The patients in this prospective observational cohort study were consecutively recruited from an ongoing randomized control trial (RCT), carried out at a regional hospital in Halmstad, Sweden [10]. Patients in the TIVA and volatile anesthesia groups in this study were recruited from standard of care groups in this ongoing RCT. In short, patients were recruited from the pain-tolerant arm, where they subsequently got randomized to either OFA or one of two protocolized routine anesthesia methods used as controls.

The project plan was published on Clinical Trials, (NCT06227143, date; 24th of January 2024), before data were accessed. Recruitment process was conducted between 1st of August 2023 and 1st of February 2024. All participants in this study gave both oral and written informed consent. The protocol was approved by the Swedish Ethical Review Authority on 2023-01-18 with Dnr 2022-07156-02. We have followed the Strengthening the Reporting of Observational Studies in Epidemiology (STROBE) guidelines when writing and reporting this study.

Inclusion criteria were elective laparoscopic surgery, age > 18 years, American Society of Anesthesiologist (ASA) class < III and ability to understand and accommodate information. Exclusion criteria were refusal to participate, pregnancy and allergy towards non-steroidal anti-inflammatory drugs (NSAIDs), as the latter was part of the multimodal concept in OFA.

### Group allocation

We included 30 patients where anesthesia was maintained with either sevoflurane and remifentanil (*volatile group)*, propofol and remifentanil by target control infusion (TCI) (*TIVA group)* or OFA. The patients were consecutively recruited from an ongoing trial where patients were risk-stratified regarding risk of acute postoperative pain using pain during venous cannulation (VCP). This risk-stratifying method has shown promise when it comes to differentiating patients into either pain-sensitive or pain-tolerant [11–13]. In order to match the OFA group with control groups, subjected to routine anesthesia methods, the patients in this study were recruited from the pain-tolerant groups since the pain-sensitive group included non GABA-ergic and non-opioid anesthetic agents that potentially would bias the pEEG regarding outcomes in this study. If patients were allocated to the pain-tolerant group they could either be randomized into receiving OFA or routine anesthesia (TIVA or Volatile), creating the three groups [10]. The anesthesia nurse and the anesthesiologist in charge were instructed to perform induction and maintenance of anesthesia using conservative measures, i.e. everything but the pEEG monitor device like blood pressure, heart rate, anesthetic drug dosage, pupillary size and skin appearance.

### Anesthesia protocol

*Total intravenous anesthesia;* Induction and maintenance of anesthesia was conducted using a TCI protocol (Marsh model) with propofol and remifentanil in conjunction with betamethasone 4 mg i.v [14]. Anesthesia was further maintained with propofol and remifentanil at the discretion of the anesthesia-provider in charge.

*Anesthesia with volatile agent;* Induction of anesthesia was conducted using propofol and remifentanil in conjunction with betamethasone 4 mg i.v. Anesthesia was maintained with sevoflurane and remifentanil at the discretion of the anesthesia-provider in charge. Remifentanil was administered using TCI-protocol [14].

*Opioid-free anesthesia;* As soon as vascular access was established, and the patient arrived in the operating theatre, an infusion of dexmedetomidine was started at 0.2 µg/kg/hour and maintained throughout the perioperative process until the patient left the PACU. General anesthesia was induced using propofol and esketamine 0.1 mg/kg bolus in conjunction with betamethasone 8 mg i.v. Anesthesia was maintained with sevoflurane (MAC 0.6-1.0) together with infusion of esketamine 0.1–0.3 mg/kg/hour and dexmedetomidine 0.2 µg/kg/hour.

### Monitoring of brain function during anesthesia and data description

**Fig. 1:**
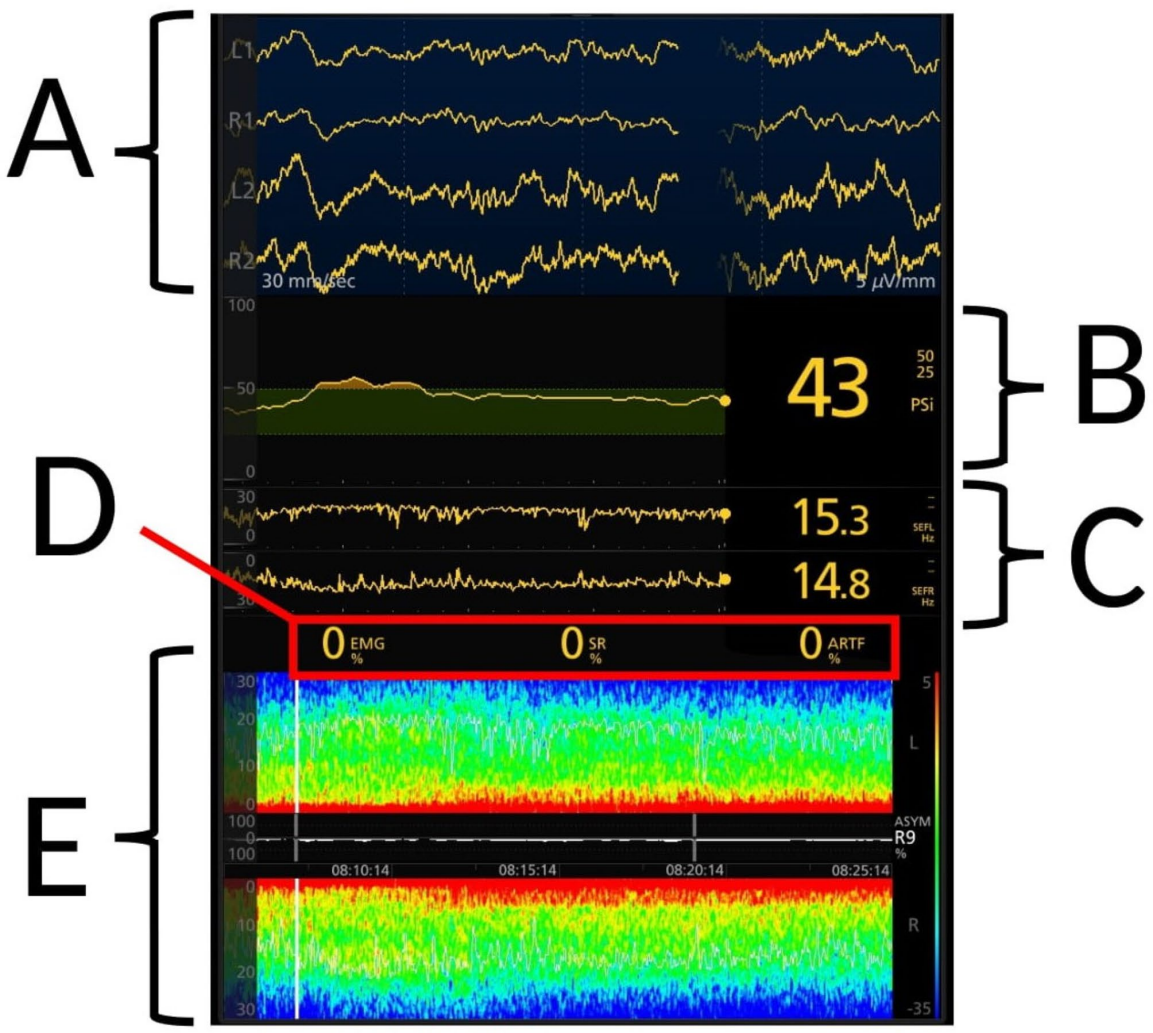
Description of Sedline Monitor indices. (**A**) The four channels of electroencephalography (EEG) representing two locations on right and left frontal lobes respectively. (**B**) Patient state index (PSI), algorithm-based quantitative index for monitoring of brain function to determine anesthesia depth during general anesthesia. (**C**) Spectral edge frequency (SEF95) containing 95% of the electric activity, also separated for left and right side. (**D**) Electromyography (EMG), which is an index over temporal muscle relaxation. Suppression ratio (SR) represents percentage of isoelectric electrical activity (+/- 0.5 µV) per minute in prefrontal and frontal cortex. Artefacts (ARTF) reflect environmental noise as artifacts, conveyed as vertical white lines in DSA. (**E**) Density spectral array (DSA) showing the power of frequencies over time as a color-scale. The upper and lower part reflects left and right frontal lobes

We used the Root^®^ and SedLine^®^ (Masimo Corporation, Irvine, CA, USA) monitor for brain function during general anesthesia. The monitor displays four channels of unprocessed EEG (two per side of the brain), PSI, left and right SEF95, suppression ratio (SR), electromyography (EMG) and DSA (Fig. 1). Patient state index is a number calculated by a Sedline algorithm reflecting the level of unconsciousness and should be between 25 and 50 for routine opioid based general anesthesia [4]. Spectral edge frequency 95 is the frequency, measured in Hz, below which 95% of the frequency-power is represented [15]. Suppression ratio (SR) is the percentage of time by which the oscillating activity is isoelectric (+/- 0.5 µV), during a one-minute epoch, in the frontal and pre-frontal cortex. A high contrast-enhancement (Multitaper) was used on all patients and DSA trends were set to – 5 dB (upper limit) and – 30 dB (lower limit) as standard. Frequency limit were set between 0 and 30 Hz. As the research question regarded a descriptive comparison between anesthesia methods, rather than individual patients, it was allowed to change contrast enhancement with the purpose of achieving the best and most representative illustrations for each individual patient at the time of data collection.

### Data collection and outcome measures

Data regarding the included patients was collected from standard hemodynamic monitoring (heart rate, non-invasive blood pressure), drug dose from infusion-pumps and the ventilator (calculated age adjusted minimal alveolar concentration).

Data regarding the brain state during general anesthesia was collected from the Sedline monitor (Fig. 1) when anesthetic steady state, after surgical incision, had been achieved for at least 20 min. The attending anesthesiologist and anesthesia nurse were blinded to the pEEG monitor. When the clinicians confirmed adequate level of unconsciousness and anesthetic steady-state, after skin incision, has been maintained for at least 20 min, quantitative data and DSA-patten was extracted. All patients were followed up with an interview regarding potential intraoperative awareness and postoperative hallucinations.

Primary outcome was the difference in mean PSI between the three anesthesia groups (TIVA vs. Volatile vs. OFA). We defined the null hypothesis as; There is no difference in mean PSI between groups.

The secondary outcome was the difference in mean SEF95 between the three anesthesia groups. The mean SEF95 used in data analysis was calculated from the right and left prefrontal cortex area (SEF95R and SEF95L) on the monitor. Finally, we used the DSA-graphs for a case derived comparison of DSA-pattern between OFA and the two other routine anesthesia groups (TIVA, Volatile).

### Statistical analysis

In a study comparing the bispectral index (BIS) with PSI during TIVA with propofol and remifentanil the mean standard deviation (SD) for PSI values at different time points was 9.1 [16]. We assumed our group means for PSI to be 30, 35 and 45 for TIVA, volatile and OFA groups respectively. Calculating power analysis for *one-way ANOVA* comparing three groups with the power of 0.8 and alpha-value of 0.05, the calculated sample size in each group was 8. We decided to include 10 patients for each group to be able to detect a difference in primary outcome.

The data was concluded to be normally distributed with a *Shapiro-Wilk test.* A *Levene´s test* was done and resulted in no significant variance between groups. As data for primary, secondary outcomes and age were of continuous origin, and groups were independent, the null hypothesis was tested using *one-way-ANOVA*.

Data is presented as mean values and standard deviation as dispersion measure. Statistical significance was set at a *p*-value of less than 0.05.

The data was analyzed using the IBM SPSS 29.0 (IBM Inc., Armonk, NY, USA) software.

## Results

### Patient characteristics

A total of 30 patients were included in the data analysis. Ten patients were allocated to each group (volatile, TIVA, OFA). There was a numeric difference in age between the groups, however, this difference was not statistically significant (*p* = 0.142) (Table 1). Statistical analysis concluded that there was no significant difference in hemodynamic parameters, during anesthetic steady state after skin incision. There was a predominance of female gender in all groups, with no difference between groups (Table 1).

**Table 1:** Descriptive data of enrolled patients

	Volatile	TIVA	OFA	*p* - value
Number of patients	10	10	10	
Age, years	55 (± 18.4)	49 (± 16.4)	65 (± 18.2)	0.142
Female gender	8/10	7/10	7/10	
Heart rate	63 (± 12.7)	62 (± 10.1)	71 (± 10.3)	0.176
Systolic blood pressure, mmHg	109 (± 18.5)	100 (± 37.7)	100 (± 17.9)	0.661
Diastolic blood pressure, mmHg	67 (± 10.0)	63 (± 11.6)	68 (± 6.4)	0.510
Patient state index	31 (± 3.9)	28 (± 3.7)	40 (± 8.5)	< 0.001*
Spectral edge frequency, Hz	12.5 (± 1.8)	11.1 (± 1.3)	16.4 (± 3.1)	< 0.001*

Oneway ANOVA comparison between groups in regard to study outcome and clinical parameters. A single asterisk (*) indicates a significant difference between study groups. The standard deviation is reported within the brackets

### Primary quantitative outcome

**Fig. 2:**
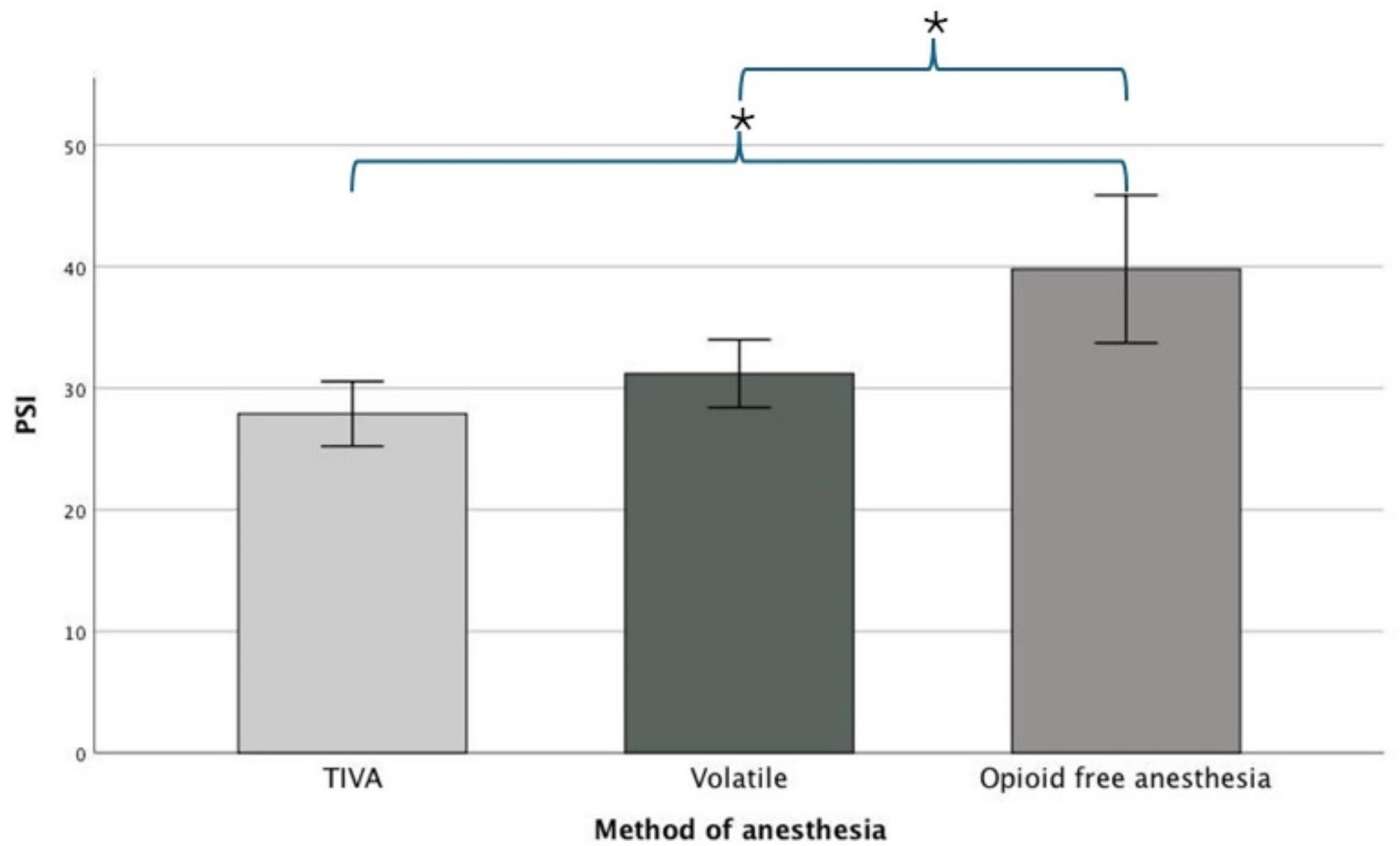
Primary outcome. Bar chart illustrating difference in mean patient state index (PSI) between total intravenous anesthesia (TIVA), volatile and opioid-free anesthesia (OFA) groups. The whiskers represent the 95% confidence interval. A single asterisk (*) indicates a statistically significant difference between groups

At the drug doses used in this study, and when anesthetic steady state after skin incision was achieved, there was a statistically significant difference in mean PSI between groups as determined by one-way ANOVA (*F* (2.27) = 11.173, *p* < 0.001). A Tukey post hoc test revealed that the mean PSI was significantly lower in TIVA group at 27.9 (SD = ± 3.7, *p* < 0.001) and volatile group at 31.2 (SD = ± 3.9, *p* = 0.007) compared to OFA group which had a mean value of 39.8 (SD = ± 8.5) (Table 1). There was no statistically significant difference in mean PSI between TIVA and volatile groups (*p* = 0.424) (Fig. 2).

### Secondary quantitative outcome

**Fig. 3:**
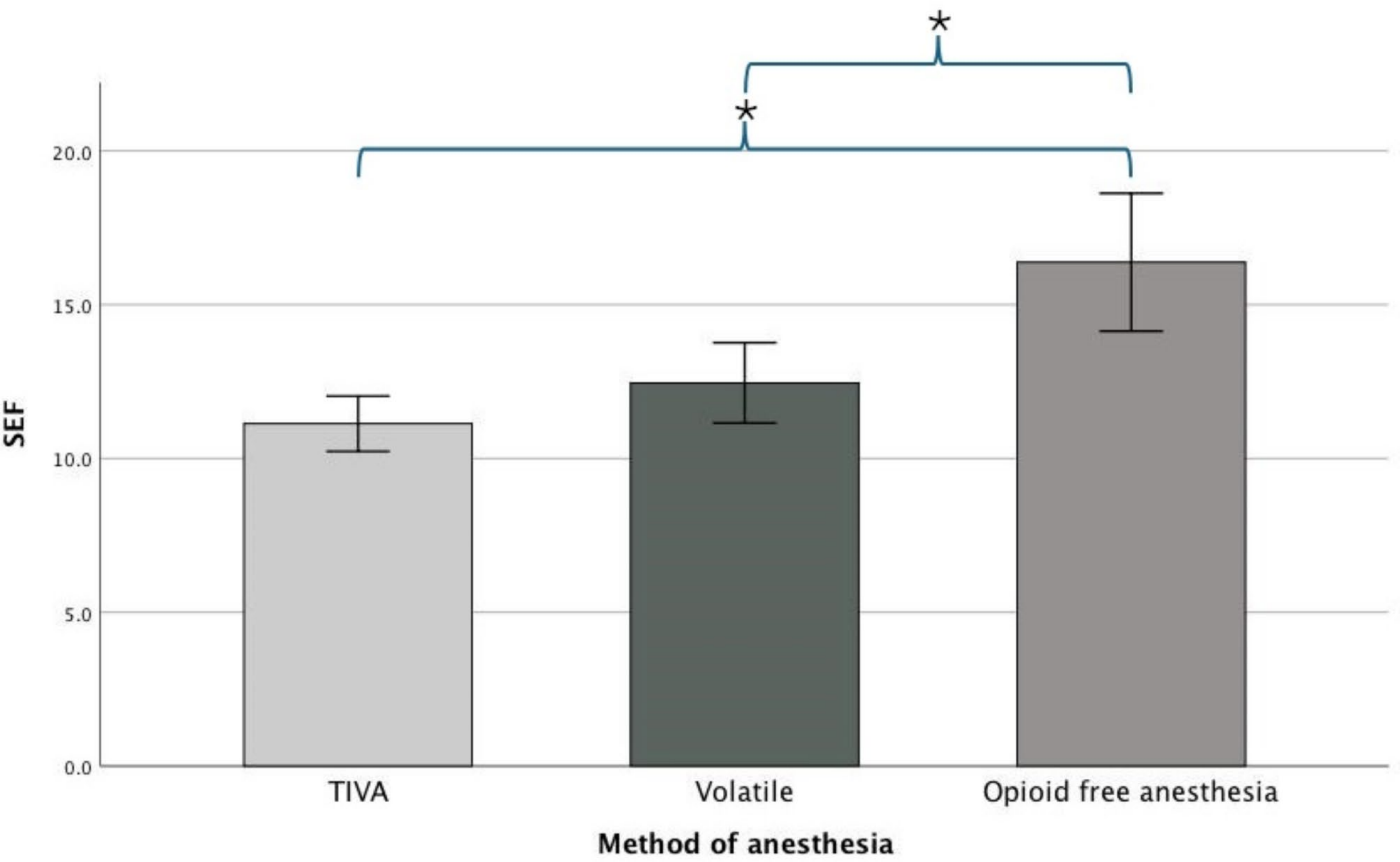
Secondary quantitative outcome. Bar chart illustrating difference in mean spectral edge frequency (SEF) between total intravenous anesthesia (TIVA), volatile and opioid-free anesthesia (OFA) groups. The whiskers represent the 95% confidence interval. A single asterisk (*) indicates a statistically significant difference between groups

At the drug doses used in this study, and when anesthetic steady state after skin incision was achieved, there was a statistically significant difference in mean SEF95 between groups as determined by one-way ANOVA (*F* (2.27) = 15.207, *p* < 0.001). A Tukey post hoc test revealed that the mean SEF95 was significantly lower in TIVA group at 11.1 (SD = ± 1.3, *p* < 0.001) and volatile group at 12.5 (SD = ± 1.8, *p* = 0.001) compared to OFA group (16.4 ± 3.1) (Fig. 3). There was no statistically significant difference between TIVA and volatile groups (*p* = 0.387) (Table 1).

None of the patients in this study had potential interference from EMG. Three patients had a SR of 4%, 6% and 6% respectively, two of which being in the TIVA-group and one in OFA-group.

### Secondary descriptive outcome

#### Opioid-free anesthesia

**Fig. 4:**
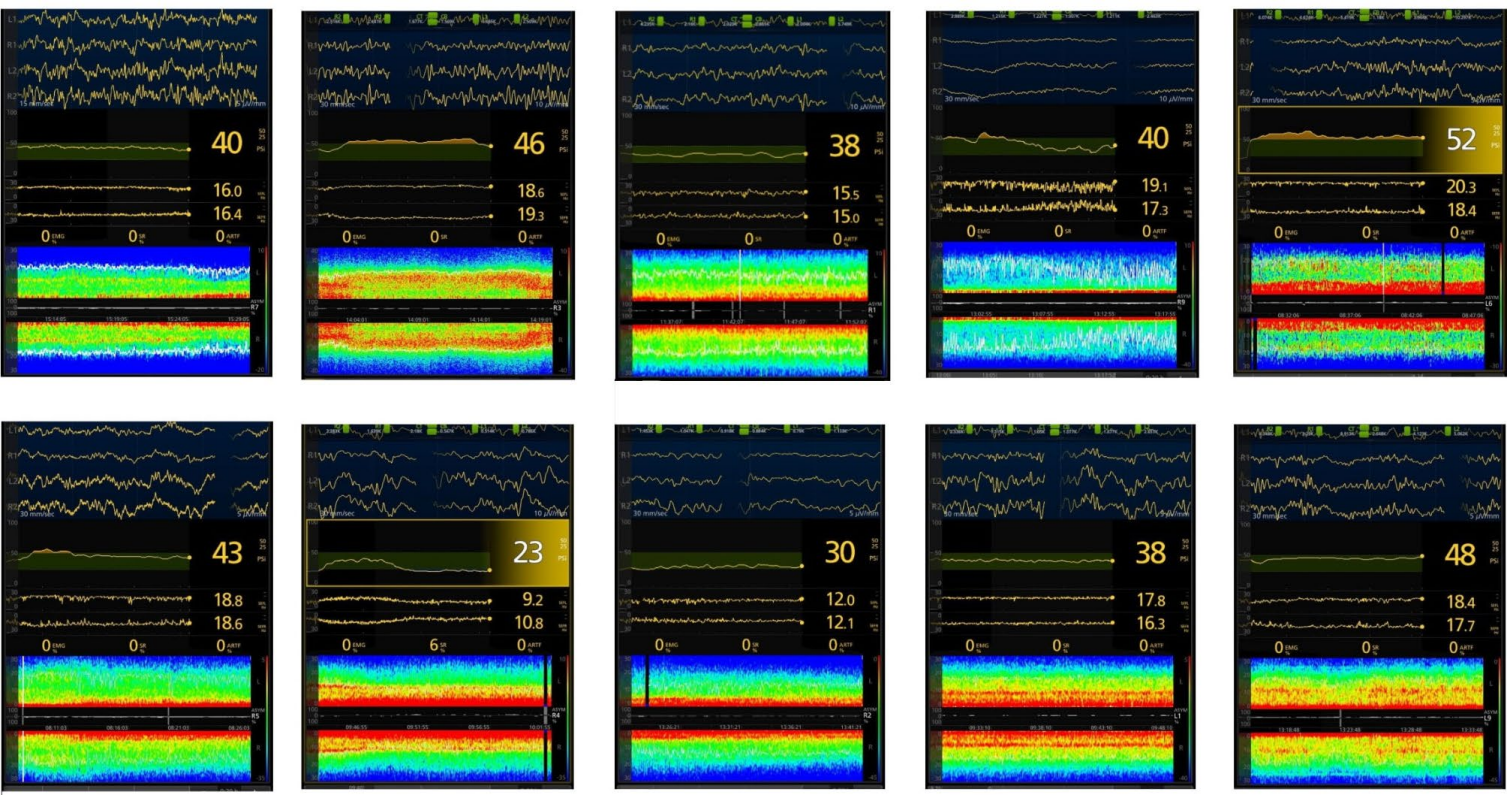
Secondary quantitative outcome. Data from processed electroencephalography and density spectral array (DSA) for all ten patients in opioid-free anesthesia (OFA) group. Patients in this figure were labeled a_1_ - j_1_ starting on top left to bottom right. All patients were considered to be in anesthetic steady state after skin incision

There was typically a different DSA-pattern compared to the routine anesthesia groups with generally a predominance of power (red) in the theta-spectrum whilst a low-powered spectrum (green) was seen in the delta to beta spectrum (Fig. 4). This was seen in most patients except for patients (g_1_), (i_1_) and (j_1_), who all exhibited a typical -alpha/delta- pattern recognized in established routine anesthesia techniques. For one patient (b_1_) there was mostly a high-power pattern in the low beta-spectrum, resembling patterns of N-Methyl-D-aspartate (NMDA)-antagonists, with a SEF95 of 19.0 and PSI of 46. For (d_1_) a similar pattern was seen as for the other patients (a_1_, c_1_, e_1_-h_1_), however there was a more widespread absence of the low-power pattern seen in the other examples.

One patient (g_1_) had a SR of 6% during anesthetic steady state after skin incision. This was a 30-year-old female undergoing robotic gynecologic surgery receiving Sevoflurane at MAC 1.0, dexmedetomidine 0.2 µg/kg/hr and esketamine 0.2 mg/kg/hr. This patient had lower than average SEF95 and PSI of 10 and 23 respectively. Another two patients (i_1_, j_1_) exhibited a more classic -alpha/delta-pattern, however with a high SEF95, 17.1 Hz and 18.1 Hz respectively, and PSI, 38 and 48 respectively. None of these patients received interfering EMG signals, that otherwise could potentially increase SEF95-values. Mean MAC for sevoflurane was 0,9 with a range of 0.8 to 1.2. No patients in this group described an experience of awareness during the intraoperative phase.

#### Total intravenous anesthesia

**Fig. 5:**
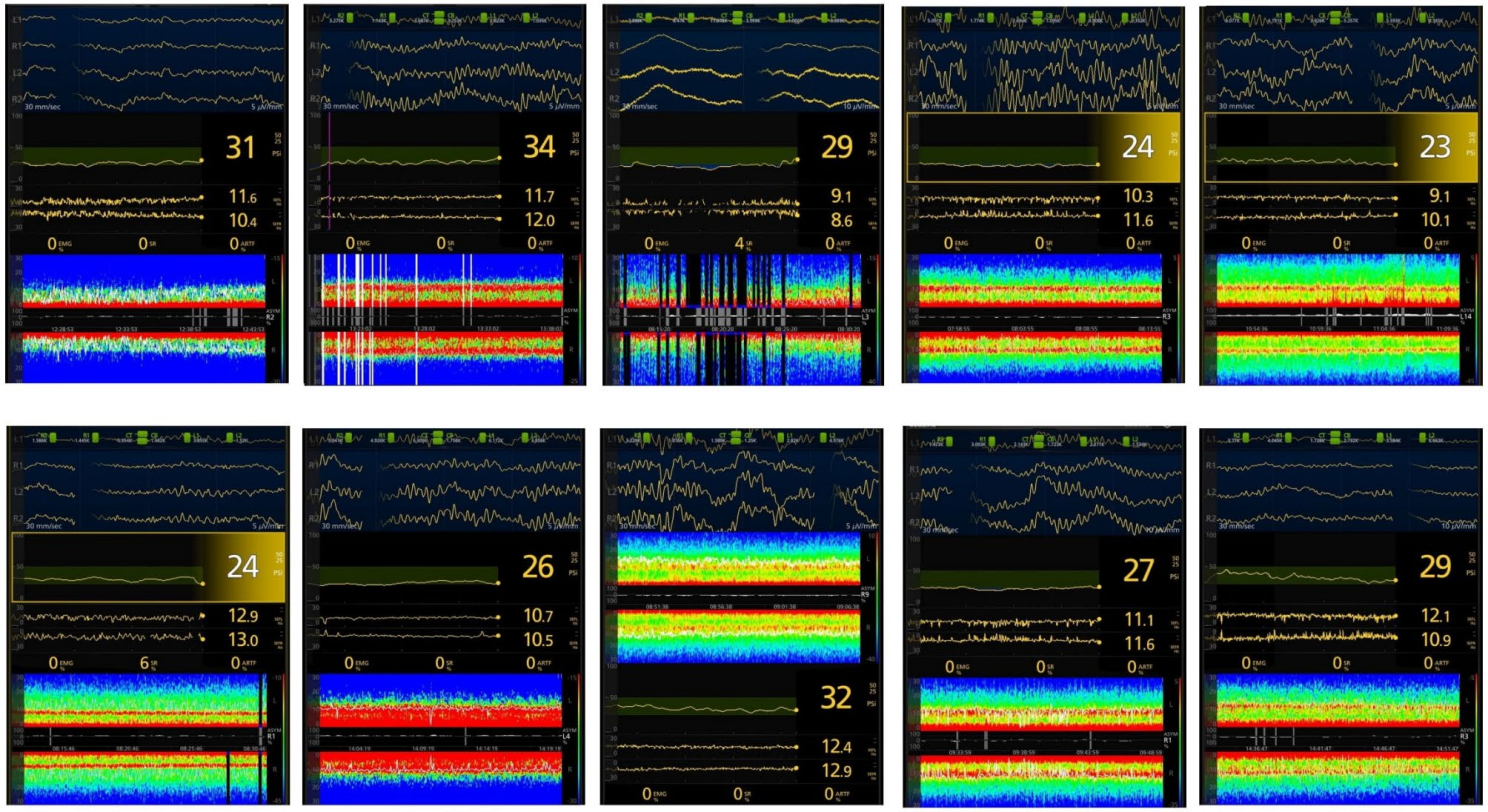
Secondary quantitative outcome. Data from processed electroencephalography of anesthesia and density spectral array for all ten patients in total intravenous anesthesia (TIVA) group. Patients in this figure were labeled a_2_ - j_2_ starting on top left to bottom right. All patients were considered to be in anesthetic steady state after skin incision

All but one patient exhibited a well-described alpha/delta-frequency pattern on DSA (Fig. 5) [6]. Patient (a_2_) had no visual frequency pattern in the alpha spectrum, but rather almost exclusively in the theta spectrum. However, when looking at the raw-EEG there is clearly a presence of alpha-waves at approximately 10–12 Hz. This patient was a 66-year-old male undergoing laparoscopic colorectal surgery with calculated serum-levels of 4.7 ng/ml (remifentanil) and 2.5 µg/ml (propofol). One patient (c_3_) had isoelectric EEG-pattern with an SR of 4%. This was a 63-year-old female undergoing robotic gynecologic surgery with an on average low calculated serum concentration of remifentanil (2.5 ng/ml) and propofol (2.5 µg/ml). Patient g_2_ had a clear theta (Hz 5–7) fill in effect although PSI and SEF95 values were not dramatically different from the other patients. This patient was 60 years old and needed a bit higher dose of Remifentanil (6.0 ng/ml) and propofol (3.0 µ/ml) for clinically reaching an adequate level of anesthesia with preserved vital parameters, blood pressure 120/70 and heart rate 75. The fill in effect could be due to higher doses of remifentanil but also partly due to a change of contrast enhancement. No patients in this group described an experience of awareness during the intraoperative phase.

#### Volatile anesthesia

**Fig. 6:**
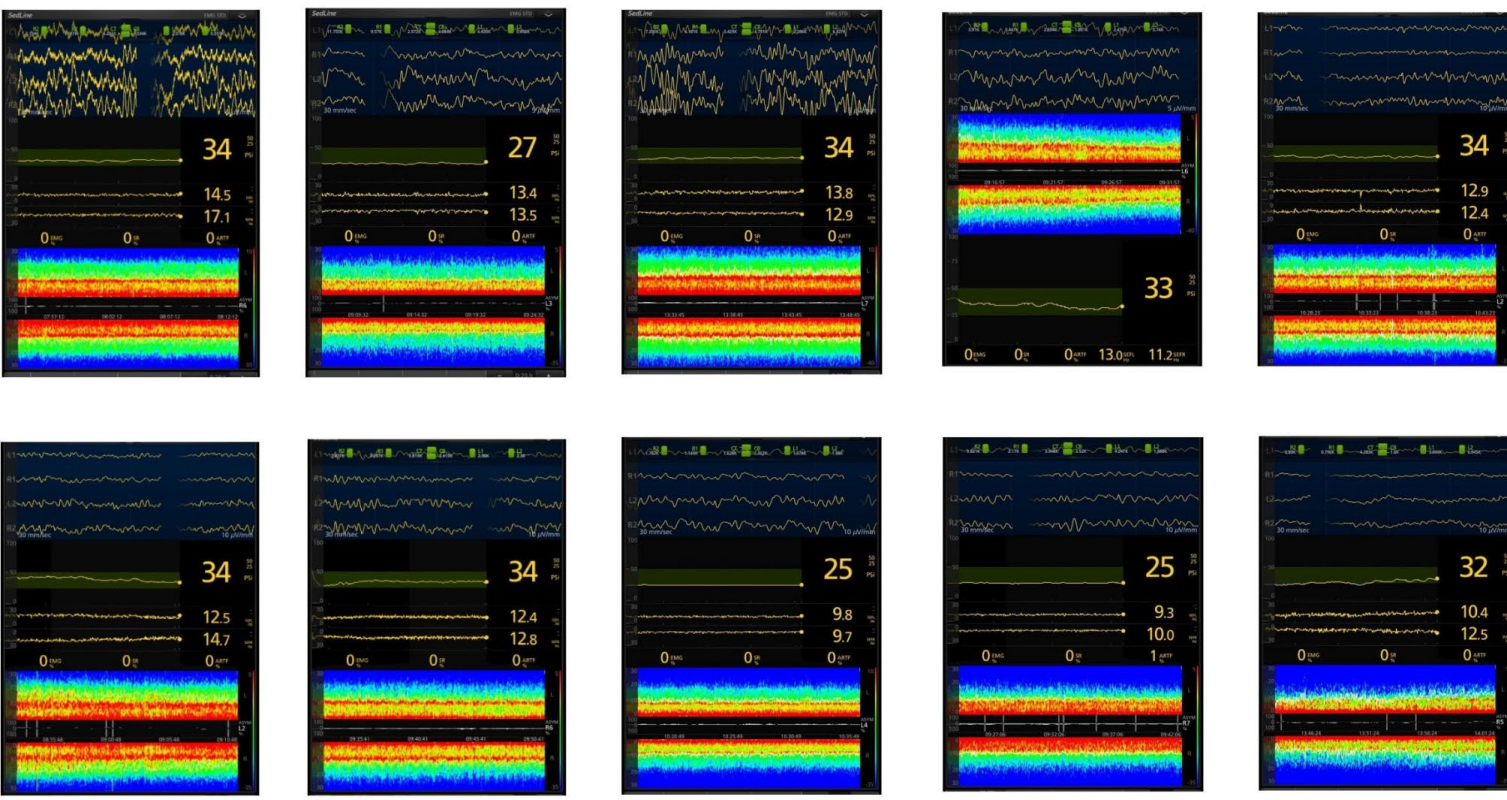
Secondary quantitative outcome. Data from processed electroencephalography of anesthesia and density spectral array for all ten patients in volatile group. Patients in this figure were labeled a_3_ - j_3_ starting on top left to bottom right. All patients were in anesthetic steady state after skin incision

All patients showed a similar DSA-pattern, more precisely high power in the alpha/delta-frequency spectrum (Fig. 6). No patient in this group had interfering EMG-activity or isoelectric EEG-activity. Mean MAC was 0.8 with a range of 0.7 to 0.9. Mean calculated serum level remifentanil was 4.7 ng/ml, ranging between 3.0 and 6.0 ng/ml. No patients in this group described an experience of awareness during the intraoperative phase.

## Discussion

In this observational cohort study, we have used DSA, SEF95 and PSI from pEEG monitoring to compare OFA with two types of routine anesthesia. Findings confirm that pEEG can be used for OFA that is mainly based on volatile anesthetic agents in combination with other non-opioid intravenous agents, all affecting the EEG-pattern [6]. We believe that it is important to validate OFA towards monitoring techniques in order to guarantee patient safety and equity when using this anesthetic modality.

In this study both PSI and SEF95 were significantly higher compared to routine anesthesia groups when using pEEG to determine brain function during general anesthesia. The OFA-protocol utilized is roughly based on modulating peripheral sensitization (Etoricoxib, paracetamol, betamethasone), NMDA-receptors (esketamine) in the spinal cord and α_2_-receptors (dexmedetomidine) affecting the descending anti-nociceptive pathways [17]. One key issue in this study is to evaluate the effect of combinations of several anesthetic agents on neurophysiologic signaling and pEEG-pattern. Earlier studies have shown that dexmedetomidine and ketamine, used separately, affect EEG-pattern and DSA in a manner that is different from volatile agents and propofol [6, 18, 19]. Also, volatile agents as main anesthetic are described to show similar EEG-patterns, but with a dose-dependent difference from propofol. To our knowledge, there are currently no published data on what to expect, regarding neurophysiologic signaling and pEEG-pattern, when combining GABA-ergic, NMDA-antagonists and alpha_2_-agonists as well as non-sedating agents such as NSAID, betamethasone and paracetamol. We believe that it is reasonable that the combination of these agents effect neurophysiologic transmission in a different manner than each for itself.

In the OFA group the mean age was 66 compared to 55 and 49 in Volatile and TIVA group respectively. Since patients in this study were included from a larger RCT, we cannot fully explain why this difference was seen if not by chance. However, statistical analysis did not result in the observed difference being statistically significant. It is well-known that age influence EEG-patterns and therefore the results in this study demand a more nuanced discussion [20, 21]. In a secondary analysis, Obert et al. sought out to evaluate the influence of patients´ age on PSI [22]. In this study anesthesia was maintained either by propofol, sevoflurane or desflurane and a linear regression model showed that PSI increased with approximately 2 points per decade and SEF increased with 0.5 Hz per decade. If using their model (PSI = 0.23 * age + 22.69) for sevoflurane a resulting mean PSI would be 33 in the volatile group and 35 in the OFA group in the current study. The delta PSI values (current study minus calculated PSI from model; 35 − 33 and 40 − 35) are 2 and 5 respectively, arguing that there is a factor other than age influencing the higher PSI values seen in OFA. Since no adjunct anesthetic was reported in the aforementioned study we hypothesize, but cannot state, that OFA rather than age is the driver of our findings. Regarding SEF95, one study demonstrated that values differed between age groups receiving general anesthesia with Sevoflurane as single agent. The highest value (16 Hz) was found in the middle age group 55 to 63 years old [23]. The younger and older patient groups who received sevoflurane had lower values, 14 and 15 Hz respectively. In the OFA group the mean SEF95 was 16,4 Hz. In this group there was one outlier with mean SEF95 of 10 Hz, which if removed from analysis raised clustered mean SEF95 for OFA-group further (17.1, SD ± 2.3). With only two patients in the aforementioned age group (55–63), reported by Kanazawa et al., we doubt, but cannot exclude, that age interfered with the higher SEF95 values reported in our study. This indicates that anesthesia providers could expect higher SEF95 values in achieving adequate anesthesia depth in OFA.

Even though maintenance of anesthesia in our OFA-protocol was conducted mainly by sevoflurane and the doses of both esketamine and dexmedetomidine were very low, we saw a different DSA-pattern and quantitative data. Most notably, frequencies in the alpha spectrum were generally missing in conjunction with a high SEF95, at a level more associated with light sedation [5]. We hypothesized that the impact of NMDA-antagonism and α_2_-agonism play an intricate role in this finding. However, it would be reasonable if DSA-pattern were to be more similar with that of volatile group since sevoflurane used in our OFA-protocol is arguably the most dominant in keeping the patient unconscious. If this hypothesis was to be true, there is a low predictability from dexmedetomidine and esketamine on their effect on pEEG since doses used in this study generally were low. We believe that earlier findings, regarding the antinociceptive synergism between esketamine and dexmedetomidine, carry great influence on our findings [24]. Furthermore, the high SEF95 in the OFA-group indicates that even at a low dose, NMDA-antagonism affects the overall picture.

In the descriptive outcome we saw a DSA-pattern predominately in the theta spectrum for the OFA group. As this might be a signature for OFA it is also possible that this is a consequence of the averagely high dose sevoflurane seen (0.9 compared to 0.8) in the OFA group in comparison to the Volatile group. Earlier research have described that changes in EEG-pattern occur as the dose of isoflurane increases beyond that which cause alpha/delta-pattern [25]. More specifically, increasing the dose of isoflurane beyond that which cause alpha/delta-pattern will canalize the oscillating frequencies in the below 4 Hz range. Another study compared EEG-patterns between sevoflurane and propofol in patients undergoing general anesthesia and found that sevoflurane had larger theta and beta oscillation power, and similar slow and alpha oscillation power [26]. In the light of this we cannot exclude the fact that sevoflurane has influenced the descriptive outcome in a dose dependent manner even though the group-based difference in MAC of 0.1 is considered small. Future studies should standardize the dose of volatile anesthetic to be able to elucidate this further.

This study adds to the perspective of making OFA a safer anesthesia method as well as ensuring that it is up to date regarding newer monitoring adjuncts. Future studies should focus on reproducing findings in this study in larger cohorts. When validated, there is a potential for research in OFA where pEEG is used as a pain prediction adjunct, a research field that has potential but up till now has carried conflicting results.

### Strengths and limitations

This study was of prospective design and enrolled consecutive patients who had been randomized to receive a certain type of anesthesia. These groups were not amended regarding this study, as seen when data analysis concluded the groups to be well matched, thus lowering the risk of selection bias. In regard to bias this design and enrollment is seen as this study’s greatest strength. We also believe that having anesthesia protocols regarding drug doses, instead of leaving treatment to the discretion of the anesthesiologist, contributed to a decreased risk of attrition bias and confounding.

Previous studies have used 8–13 Hz as the optimal SEF to achieve general anesthesia [27]. With mean SEF-levels of 12.5 Hz for the volatile group and 11.3 Hz for the TIVA group we have kept within the higher span in our study. Despite that we see a clear difference in the OFA-group with SEF levels of 16.4 Hz. This might of course differ a bit depending on the combination of drugs used in the OFA protocol but one should expect a higher level of SEF.

The limitations in this study are several. The patients in the study were recruited as part of an ongoing RCT, including only elective laparoscopic surgery, affecting generalizability. This is also the case with the study-design being limited to one center with its own protocol for OFA. However, we believe that laparoscopic surgery is an excellent routine procedure with elements seen in many types of modern surgical procedures, which challenges the anesthesia team when determining anesthesia depth and nociception. The small sample size, calculated only for PSI, is probably insufficient to draw clear conclusions about DSA-pattern in OFA. We included patients who were risk-stratified as being pain-tolerant, which is a step towards a more differentiated cohort. This was one of the main purposes in the other clinical trial, from which patients in this study were recruited [10]. However, this could potentially affect the generalizability of the result as patients in this study might have a different neurophysiologic response to anesthetic agents.

Even though findings in this study are compelling, we advise readers to observe caution before implementing them into clinical practice as we believe more high-quality studies need to be conducted on this topic.

## Conclusion

Findings in this study suggest that OFA affects both quantitative indices and DSA-pattern in pEEG. More specifically, SEF95 and PSI can be expected to be higher compared to other opioid based routine anesthesia methods. Furthermore, DSA-pattern for OFA showed a high power in the theta frequency spectrum, with low power in higher frequency spectrums. More studies are needed in order for these findings to have more robust implications.

## Data Availability

Data is provided within the manuscript or supplementary information files.

## Abbreviations

ARTF: Artefacts
ASA: American Society of Anesthesia
BIS: Bispectral index
EEG: Electroencephalography
EMG: Electromhyography
MAC: Minimal Alveolar Concentration
NMDA: N-Methyl-D-Aspartate
NSAID: Non-steroidal anti-inflammatory
OFA: Opioid-free anesthesia
PACU: Post-anestheisa care unit
pEEG: Processed Electroencephalography
PSI: Patient State Index
RCT: Randomized Control Trial
SEF: Spectral Edge Frequency
SD: Standard Deviation
SR: Suppression Rate
TIVA: Total Intravenous Anesthesia
TCI: Target Control Infusion
